# The Heritage of Health

**Published:** 1923-05

**Authors:** Mabel K. Wallace

**Affiliations:** *Hon. Secretary*. 128 Pentonville Road, N.1


### CORRESPONDENCE.

[Correspondence on all subjects is invited, but we cannot be responsible for the opinions expressed by our correspondents, who must give name and address as a guarantee of good faith, but not necessarily for publication. Correspondents are reminded that conciseness greatly facilitates early insertion.]

### The Heritage of Health.

*To the Editor of* THE HOSPITAL AND HEALTH REVIEW.

SIR,—May I beg the hospitality of your influential columns on behalf of the Women's League of Service for Motherhood, a patriotic organisation which is doing national work? The League has for its objects the safeguarding of working-class mothers from lack of proper food, the safeguarding of their children from being born ill-nourished, and the safeguarding of the children when born from being half-starved or fed on wrong food. Another important work undertaken by the League is to teach to the well-intentioned but very ignorant young mothers how properly to treat their babies.

By doing these things the League is preventing the mothers from doing their work badly, and giving the country a race which is not fit to carry on our traditions bravely. In the necessitous areas of London there are a very large number of mothers who are half-starved, both before their children are born and afterwards. What that means to the child it does not need a doctor to tell. We see the proofs of it every day in the streets in puny, miserable creatures who should be bright and bonny; in the special schools where the weakly intelligent are trying to lessen their handicap for the battle of life; in the hospitals, where the children suffer from sickness which need not be; in homes for incurable children, who are punished for the disabilities of their mothers.

Thus is the future of the race jeopardised. To combat it the League provides daily dinners for mothers until their children are nine months old. This at least gives the child a first chance of health and strength of body and mind. Our experience is that a month of this treatment makes a marvellous difference. The women wake up and take an interest in the world and in their babies, and the home is given back an alert home-maker where before was a don't-care drudge. The dining rooms, which are situate in the midst of terribly squalid neighbourhoods, are pictures of brightness and cleanliness, and the very best kind of social service is intelligently and diligently undertaken daily among the women, both in the dining rooms and in their own homes. Infant care, hygiene, and little health talks are the natural consequences of such visits.

This work on behalf of the helpless and half-starved in the hunger zones of London should appeal to all who perceive the sacredness of little children. We can never do anything better than to render now whatever service we are able to render to these mothers and their babies. That the mothers appreciate the care and good food there is no doubt, and, what is better still, they come to understand the importance of the proper development of their children, and to realise that it is only through the well-being of the mothers that this is possible. It is a campaign against preventable disease and misery, incapacity and poverty of race.

Voluntary helpers are needed to help this national undertaking, which is endeavouring to restore to England the sacrifices she made during the Great War. I will gladly answer any inquiries.

MABEL K. WALLACE,

128 Pentonville Road, N.1. *Hon. Secretary.*

